# Effect of fire factors (smoke, ash, charcoal and heat) on seeds of plant species

**DOI:** 10.1016/j.mex.2022.101679

**Published:** 2022-03-26

**Authors:** Óscar Cruz, Sheila F. Riveiro, Mercedes Casal, Otilia Reyes

**Affiliations:** BIOAPLIC Group. Area of Ecology. Department of Functional Biology, Faculty of Biology, University of Santiago de Compostela, Santiago de Compostela 15782, Spain

**Keywords:** Fire treatments, Incubation conditions, Scarification methodology, Seeds Germination, Viability test

## Abstract

The effect of the main fire factors (smoke, ash, charcoal and heat) can influence the germination of species through their seeds. Hence, a methodology has been devised in order to have a common protocol for those who work in this area and serve as a valuable tool to compare different species that can be beneficial or detrimental to the conditions of a forest fire. Moreover, the methodology was completed with a check of the viability of the seeds before starting the germination process and another viability test with the non-germinated seeds (dormant or dead) after carrying out the germination test. In this way, the study of the main factors of fire, in combination with the study of the viability of the seeds, can be very useful to know the reproductive behaviour of herbaceous, shrub and tree species in a forest fire scenario. In addition, from the data obtained during the germination process of the seeds, a series of explained parameters can be obtained to better interpret the data acquired from the laboratory experiment and subsequent comparison. For all these reasons, the study of the germination of species in laboratory conditions can help us understand how they behave in the field.

## Graphical abstract

**Figure:**
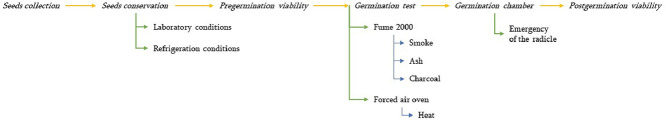

Specifications table

**Table:** 

Subject Area:	Agricultural and Biological Sciences
More specific subject area:	*Fire Ecology*
Method name:	*Fire treatments*
Name and reference of original method:	*Reyes, O., Casal, M. Germination behaviour of 3 species of the genus Pinus in relation to high temperatures suffered during forest fires (1995) Ann. For. Sci 52(4) 385–392. 10.1051/forest:19950408* *Reyes, O., Casal, M. Can smoke affect the germination of* Pinus sylvestris*, P. nigra, P. uncinate and P. pinaster? (*2006*). Forest Ecology and Management 234 S184. 10.1016/j.foreco.2006.08.303* *Reyes, O., Casal, M. Seed germination of Quercus robur, Q. pyrenaica and Q. ilex and the effects of smoke, heat, ash and charcoal (2006). Annals of Forest Science 63(2) 205–212. 10.1051/forest:2005112*
Resource availability:	*N.A.*

## Method details

Fire is a factor that causes great damage to plant communities. That is why it is necessary to study how fire, through its most important factors (smoke, ash, charcoal and heat) can influence the germination aspects of plant species. After a forest fire, plants have two main methods of regeneration: resprout and germination. It is necessary to join efforts to obtain comparable results between species throughout the world and for this reason it is necessary to use the same methodology. Currently, there is a great dispersion in the methods used to carry out germination tests in relation to fire. This lack of unanimity in the criteria to be used has led us to propose a standardized protocol. This protocol focuses on the germination pathway and describes the methodology we apply to determine the effect of different fire factors on the germination of the seeds of different plant species. The fire factors analysed are heat, smoke, ash and charcoal [1,6,21]. Table 1.

**Table 1:** Treatments carried out on the seeds collected from the different species of interest. Smoke can be obtained from the studied species or from abundant species in the study area.

Heat	Smoke	Ash	Charcoal
80 °C- 5 min	5 min	1	Studied species
80 °C- 10 min	10 min	2	*Ulex europaeus*
110 °C- 5 min	15 min	3	–
110 °C- 10 min	–	4	–
150 °C- 5 min	–	5	–
150 °C- 10 min	–	–	–
200 °C- 5 min	–	–	–
200 °C- 10 min	–	–	–

The first step of this process is to obtain the seeds of the plant species of interest. The seeds must be collected in the field during the fruiting period of the plants. Once the seeds are collected, they are released and kept until the treatments are carried out. Depending on the species used, the seeds can be stored in paper bags under laboratory conditions, at approximately 25 °C, or in the refrigerator at 4 °C. Generally, seeds of species in the families Leguminosae and Cistaceae and other species with hard seed coats do not need cold storage. However, the Fagaceae, Betulaceae and Pinaceae are preserved for long periods only if they are cold. Other species are more sensitive, for example, Salicaceae seeds, lose viability in laboratory conditions in 15 days and to avoid loss of viability, it is recommended to freeze the seeds. A visual selection of the seeds should be made, discarding those that are damaged and those that have not completed their formation and / or maturation.

### Viability test

Before starting the study of fire factors, seed viability should be checked. This is assessed through the tetrazolium test, which consists in using a solution of 2,3,3-triphenyl tetrazolium chloride salt (colourless) as an indicator of the reduction processes that occur in living cells. Seeds are imbibed in this solution and the living tissues convert this salt into a compound called formazan (red colour) through oxidation–reduction processes in the respiration processes. The resulting staining of living tissues allows us to visually discriminate the living and dead parts of a seed.

A 1% solution of tetrazolium salt in distilled water is used. Therefore, we dilute 1 g of salt in 1 L of distilled water. Once the tetrazolium solution has been prepared, the seeds to be studied are prepared. The seeds of most of the well herbaceous or shrubby species that do not have a hard cover are deposited directly on the Petri dishes. To guarantee the representativeness of the sample, 5 replications are made. Each replica consists of a Petri dish, 9 cm in diameter, with 25 seeds. In hard-coated species, such as legumes, they must be scarified previously, without damaging the embryo to facilitate the entry of the solution into the seed. This scarification must be done carefully, by using a scalpel to cut a small part of the seed on the opposite side to where the embryo is. If the seed is small, it should be done under a magnifying glass.

15 ml of the prepared solution are poured into each replicate and kept in the dark for 24 h, since the light would cause the solution to oxidise before the seed was soaked. In some species, such as legumes, although the entry of the solution into the embryo is facilitated by scarification, the staining of living structures may not be seen until after 48 or 72 h. Furthermore, this stain is incorporated into living tissues and is stable.

Subsequently, from the data obtained from the tetrazolium test, it is possible to calculate the percentage of seeds sown in the Petri dishes that remain viable at the end of the germination test [8].

### Germination test

After checking the pre-germination viability, the Petri dishes are prepared. They are 9 cm in diameter with two filter papers in each of the dishes where the seeds will be sown without scarifying. For each species and treatment, 5 Petri dishes with 25 seeds / dish are established since it can be useful for the statistical comparison of the treatments, in addition to following the criteria established by ISTA standards [13]. The control treatment consists in 5 replicates with 25 seeds/dish irrigated with distilled water.

**Fig. 1:**
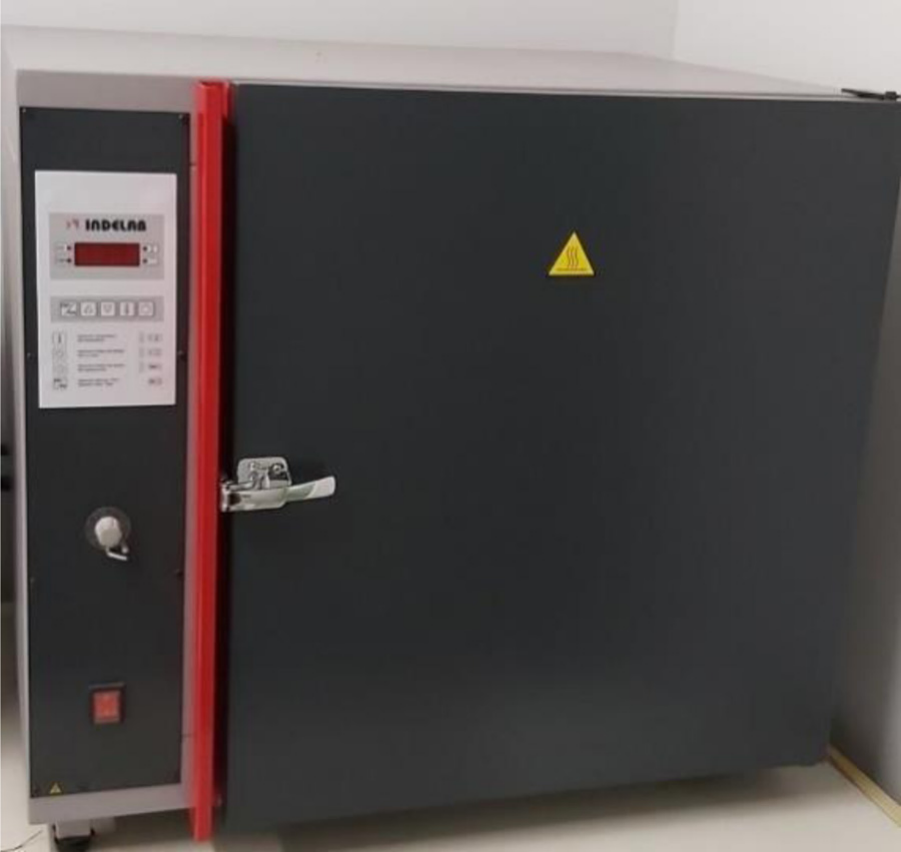
Forced air oven IDL-FI-120.

Once the dishes are prepared, the selected fire factors are applied. All fire factors are applied separately. To simulate the heat that seeds can experience during wildfires, a forced air oven (IDL-FI-120) is used to apply thermal shocks to the seeds in dry conditions (Fig. 1). The tested temperatures are 80 °C, 110 °C, 150 °C and 200 °C and the exposure times are 5 and 10 min for each temperature. Temperatures and exposure times correspond to those registered at different soil depths during forest fires and experimental burns by Auld and O'Connell [2], Bradstock and Auld [3], in Mediterranean ecosystems of southeastern Australia, by Dunn and DeBano [11], in chaparrals of California, by Trabaud [23] in French garrigues and Díaz-Fierros et al. [10], in shrublands of northwestern Spain.

**Fig. 2:**
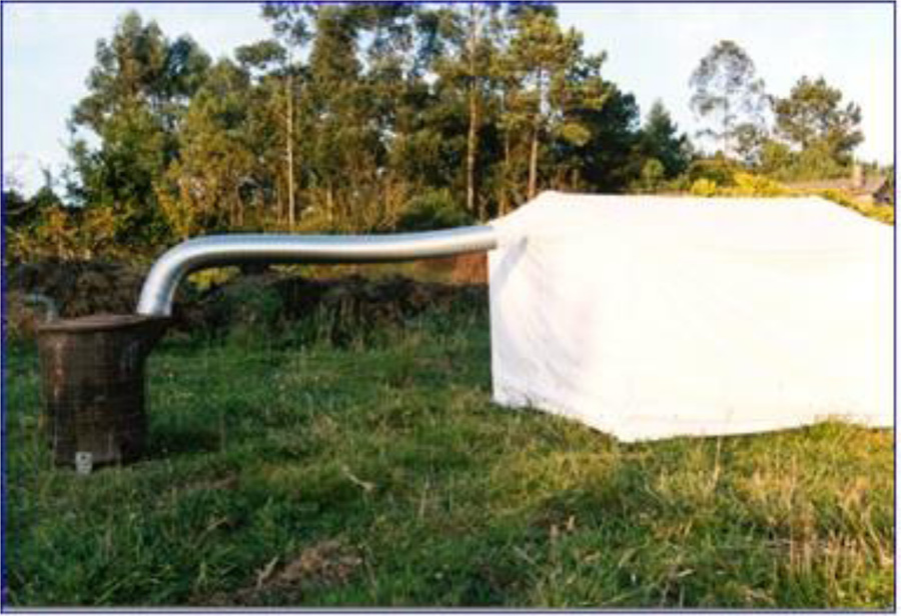
“Fume 2000” applicator.

Smoke treatments are carried out with the “Fume 2000” applicator (Fig. 2) [4] based on the methodology proposed by de Lange and Boucher [9]. The Fume 2000 device consists of a smoke generator, a cooling tube and a 2.5 m^3^ chamber that acts as a smoke receiver. The smoke circulates from the smoke generator through the cooling tube to the chamber. This system is used so that the smoke enters the chamber at room temperature, and that the effect of smoke can be perfectly isolated from the effect of heat [19]. When the smoke saturation conditions are reached inside the chamber, the seeds are introduced inside and remain there for 5, 10 and 15 min [20].

Ash and charcoal are obtained from the total and partial combustion, respectively, (approximately 20 min) of dry material (mainly branches and leaves) of the studied species. To obtain the smoke, dry biomass is mixed with green biomass. The green biomass provides more smoke, and the dry biomass ensures that the combustion does not stop during the execution of the treatment. The ash is separated from the charcoal with a 0.4 mm test sieve and the charcoal from the rest of the carbonized material with a 2.1 mm test sieve. The amounts of ash applied to Petri dishes are multiples of the amount of ash recorded in an experimental burning carried out by Soto et al. [22], in an Atlantic scrub in SW Europe. The ash treatments applied are 5, Ash 1 (0.027 g Petri dish-1, 43.5 kg ha-1), Ash 2 (0.055 g Petri dish-1, 87 kg ha-1), Ash 3 (0.11 g Petri dish- 1, 174 kg ha-1), Ash 4 (0.275 g Petri dish-1, 435 kg ha-1) and Ash 5 (0.55 g Petri dish-1, 870 kg ha-1). The charcoal treatment applied to the seeds is based on the amounts collected by Ohlson and Tryterud [14], after an experimental fire carried out in the boreal forests of Scandinavia and in previous works used by Reyes and Casal [17]. This quantity is 0.26 g Petri dish-1, (411 kg ha-1). In addition to a charcoal treatment with the study species, a charcoal treatment is carried out with that species contributing more biomass in the reference ecosystems. We use the shrub species *Ulex europaeus* L., since it is the most abundant shrub species in the understory of forest ecosystems in northwestern Spain [15,16] and from much of Atlantic Europe. Subsequently, the amounts of ash and charcoal are placed in the respective dishes previously prepared as well as marked and the seeds are sown on them.

Immediately after the application of each treatment, each Petri dish is irrigated with 5 mL of distilled water and in subsequent days with the amount of water necessary to keep the seeds moist and allow germination over time. The seeds are incubated in a phytotron germination chamber (Climas AGP890) with a photoperiod of incubation of 16 h of light at 24 °C and 8 h of darkness at 16 °C [18,20] to simulate the photothermoperiod which is typical of the northwest of the Iberian Peninsula during the summer. In other regions, this photoperiod should be adapted to that of the area to be studied at the time most germination occurs. Germination is controlled three alternate days a week until germination is complete (several days without germinated seeds in all treatments). The emergence of 1 mm of radicle is the criterion used to determine whether a seed had germinated [5]. Each time a seed germinates, it is recorded and removed from the Petri dish.

Combined treatments of several fire factors can also be done, for example [7]:

- SCAH: Smoke 10 min + Charcoal of studied specie + Ash 1 + 80 °C, 10 min.
- SH: Smoke 10 min + 80 °C, 10 min.
- CA: Charcoal of studied specie + Ash 1.

In combined treatments, it is recommended to apply heat first, then smoke, and finally ash and charcoal.

Once the seed germination period is over, post-germination viability test is applied to the seeds remaining in the ungerminated dishes. First, the seeds that have a state of putrefaction, smell bad or that can be easily destroyed by light pressure are discarded and recorded as dead. The rest of the seeds are then transferred to clean Petri dishes and the tetrazolium test is carried out to check if the seeds that did not germinate are dormant or dead.

Consequently, from data collected during the germination period of the seeds of the studied species, the following results can be extracted:

• Percentage of pre-germination viability is obtained by calculating the number of viable seeds divided by the total number of seeds per Petri dish (25 seeds) and multiplied by 100.
  $$\%PreV=\frac{number\;of\;viable\;seeds}{total\;number\;seeds\;Petri\;dish\;\left(25\right)\;}*100$$
• Germination percentage is calculated from the number of seeds germinated during the germination process, divided by the total number of seeds per Petri dish (25 seeds) and multiplied by 100.
  $$\%G=\frac{number\;of\;germinated\;seeds}{total\;number\;of\;seeds\;Petri\;dish\;}*100$$
• T_50_ or average germination rate: average time it takes to produce 50% of total germination [12]:
  $$T_{50}=\frac{\left[\left(N/2\right)-N_{1})*\left(T_{2}-T_{1}\right)\right]}{N_{2}-N_{1}}+T_{1}$$

Where *N* = final percentage of germinated seeds, N_1_ = percentage of germinated seeds below N/2, N_2_ = percentage of germinated seeds above N/2, where N_1_<*N*/2<N_2_; T_1_ = number of days corresponding to N_1_, and T_2_ = number of days corresponding to N_2_.

• Temporal distribution of germination: cumulative sum of germination produced during the germination period until its end, where each day what is produced that day plus the previous days is added.
  $$\mathrm{TD}G_{1}=N_{1};\;\mathrm{TD}G_{2}=N_{1}+N_{2};\mathrm{TD}G_{3}=N_{1}+N_{2}+N_{3}\cdots$$

Where N is the number of germinations of each day (N_1_, N_2_, N_3_…).

^1^Temporal distribution of germination or daily germination frequency is usually represented graphically.

• Post-germination viability percentage is obtained by calculating the number of viable seeds after the germination process divided by the number of seeds that remain ungerminated after the germination process and multiplied by 100.

$\%PostV=\frac{number\;of\;viable\;seeds}{total\;number\;of\;seeds\;Petri\;dish\;}*100$
